# Supplementary material for: Skillful multiyear prediction of marine habitat shifts jointly constrained by ocean temperature and dissolved oxygen
Source: Nat Commun. 2024 Jan 31;15:900. doi: 10.1038/s41467-024-45016-5 (PMC10831107; doi:10.1038/s41467-024-45016-5)
Supplement: Supplementary file 1 — Supplementary Information [file 41467_2024_45016_MOESM1_ESM.pdf]

Supplementary Information

Skillful Multiyear Prediction of Marine Habitat Shifts Jointly Constrained by Ocean Temperature and Dissolved Oxygen

Zhuomin Chen[1*], Samantha Siedlecki[1], Matthew Long[2], Colleen M. Petrik[3], Charles A. Stock[4], and Curtis A. Deutsch[5]

[1]University of Connecticut, Department of Marine Sciences, Groton, CT 06340

[2]Climate & Global Dynamics Laboratory, National Center for Atmospheric Research, Boulder, CO 80305

[3]Scripps Institution of Oceanography, University of California San Diego, La Jolla, CA 92037

[4]Geophysical Fluid Dynamics Laboratory, NOAA, Princeton University, Princeton, NJ 08540

[5]Department of Geosciences/High Meadows Environmental Institute, Princeton University, Princeton, NJ 08540

[*]Corresponding author: zhuomin.chen@uconn.edu

Contents of this file:

Supplementary Figures 1-9

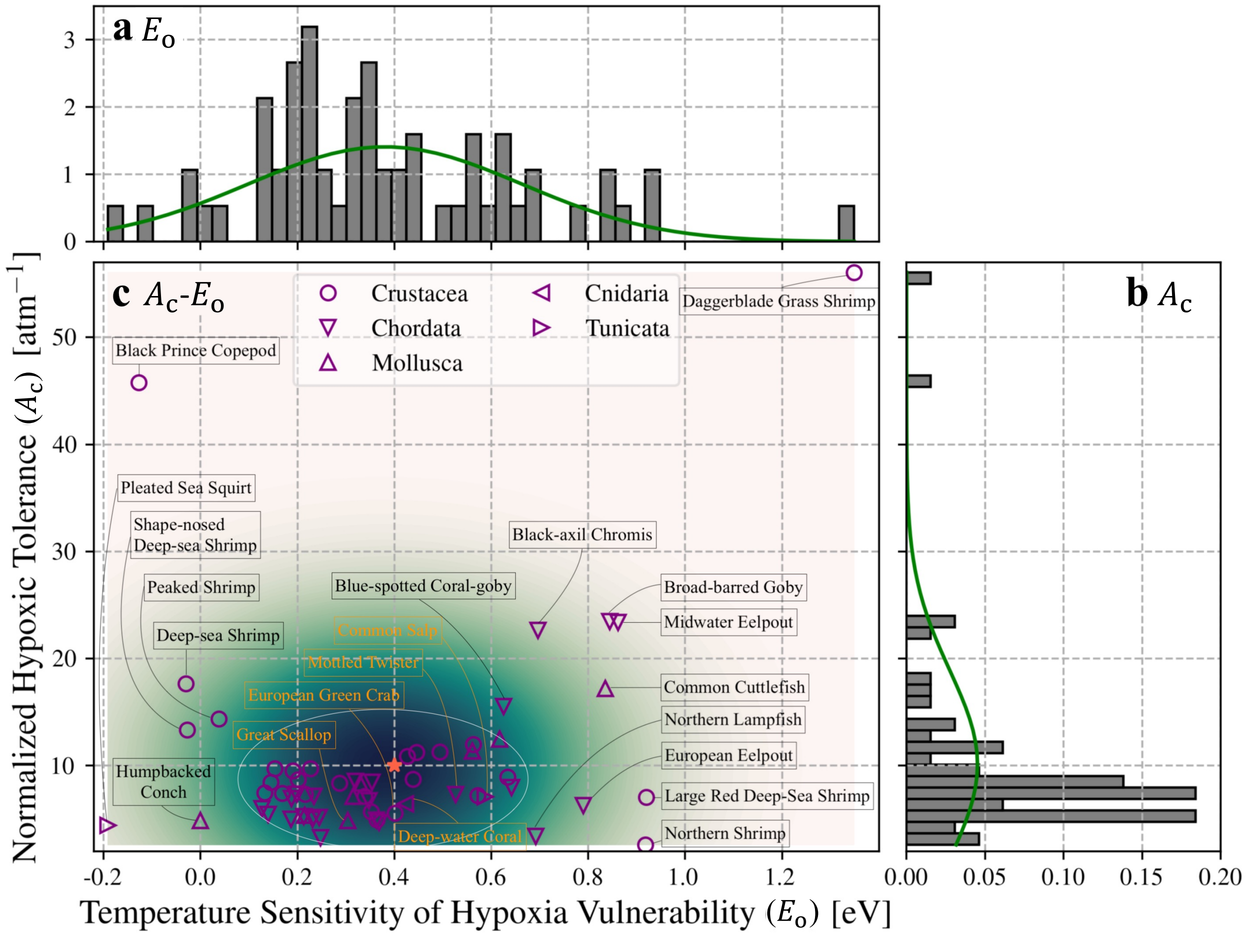

Supplementary Figure 1. Metabolic traits distribution based on the database from ref.[1]. **a-b** Histograms of the two metabolic traits - temperature sensitivity of hypoxia vulnerability (E_o; **a**) and normalized hypoxic tolerance (A_c; **b**), and their fitted probability density functions (PDFs; green curves). **c** The A_c-E_o trait space with purple hollow markers representing observations sorted by Phylum and the fitted two-dimensional PDF distribution (represented by color shading). The orange star represents the medium values of the two traits (A_c=10 atm^{-1}, E_o=0.4 eV). Common names of species with extremely low and high E_o traits are indicated on the panel. The rest species (E_o between 0.1 and 0.65 eV, A_c between 3 and 15 atm^{-1}; located closely within the white circle) include different deep-water shrimps (e.g., purple, elegant, northern brown, and white-leg shrimps), crabs (e.g., Chesapeake blue crab, Atlantic rock crab, European green crab, and common spider crab), squids (longfin inshore squid and Humboldt squid), lobsters (western rock lobster and California spiny lobster), cods (Atlantic cod and Greenland cod), basses (black sea bass and striped bass), prawns (valuable blunt-tail prawn, common American prawn, and California spot prawn), Pacific oyster, Atlantic salmon, short-nose sturgeon, Japanese eel, summer flounder, lumpsucker, scallop, twisters, common salp, red drum, small-spotted catshark, blue lantern fish, cunner, common dentex, sheep-head bream, blue-spotted coral-goby, reef-building deep-water coral, chambered nautilus, and common octopus. Only one species' common name of each Phylum (within this white circle) is indicated in panel **c** (in orange) for clear visualization. Source data are provided as a Source Data file.

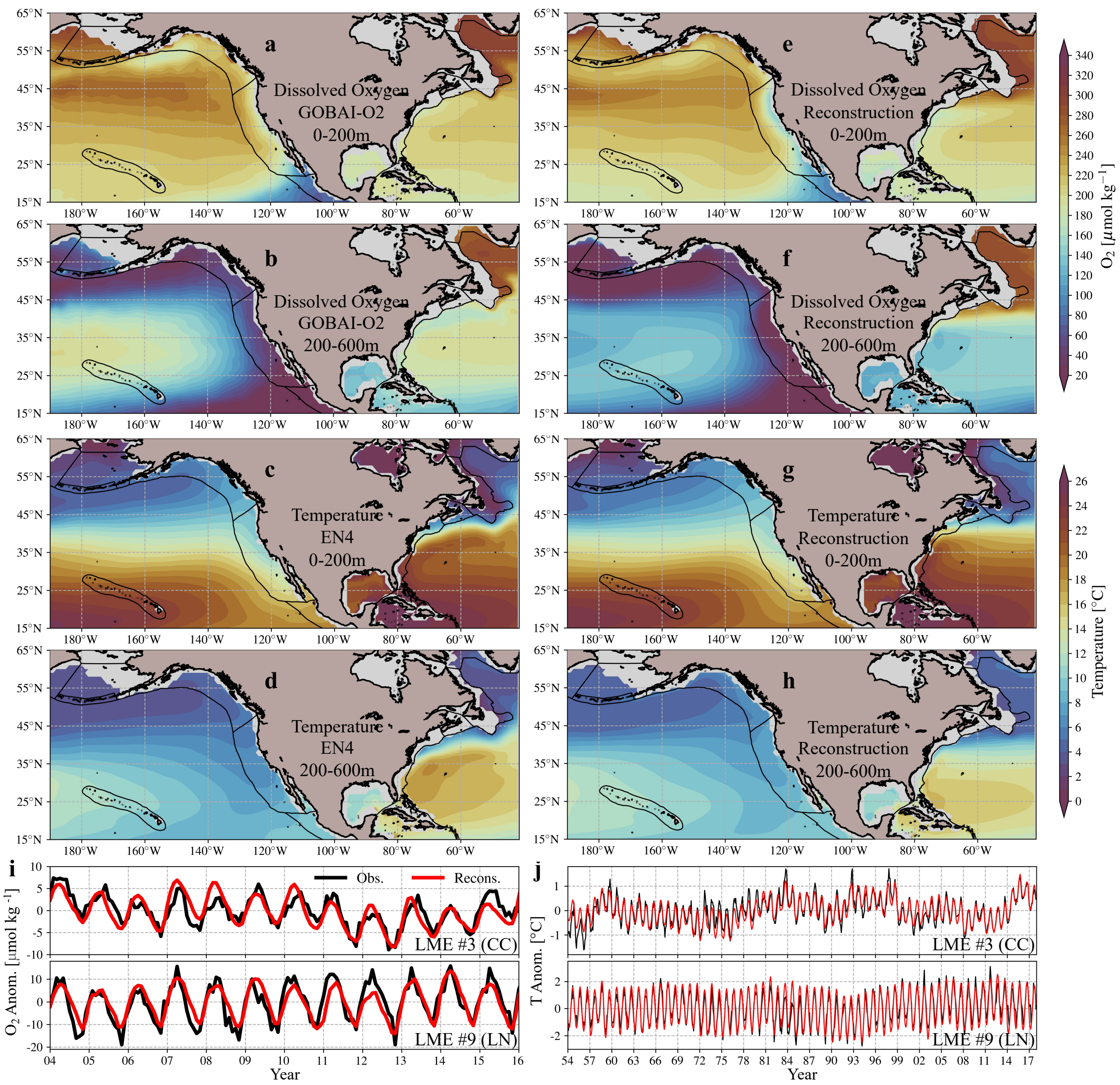

Supplementary Figure 2. The reconstruction model evaluation in dissolved oxygen (O_2) and temperature (T) compared against observation products of O_2 from the GOBAI-O2 product[2] (2004-2015) and T from the EN4 version 4.2.2 product[3] (1954-2017). **a-h** Climatological averages of O_2 (**a-b** and **e-f**) and T (**c-d** and **g-h**) at the upper-200 m (**a**, **c**, **e**, and **g**) and deeper 200-600 m (**b**, **d**, **f**, and **h**) layers between observation products (**a-d**) and the model reconstruction (**e-h**). Boundaries of the Large Marine Ecosystems (LMEs) are plotted as black solid lines in each panel. **i-j** Monthly mean time series comparison of O_2 (**i**) and T (**j**) anomalies (with the climatological averages removed) in two observation-abundant LMEs - the California Current (CC; LME 03) and Labrador-Newfoundland (LN; LME 09), at the upper-200 m layer. The black line in each panel represents the observation product, and the red line represents the model reconstruction. Source data are provided as a Source Data file.

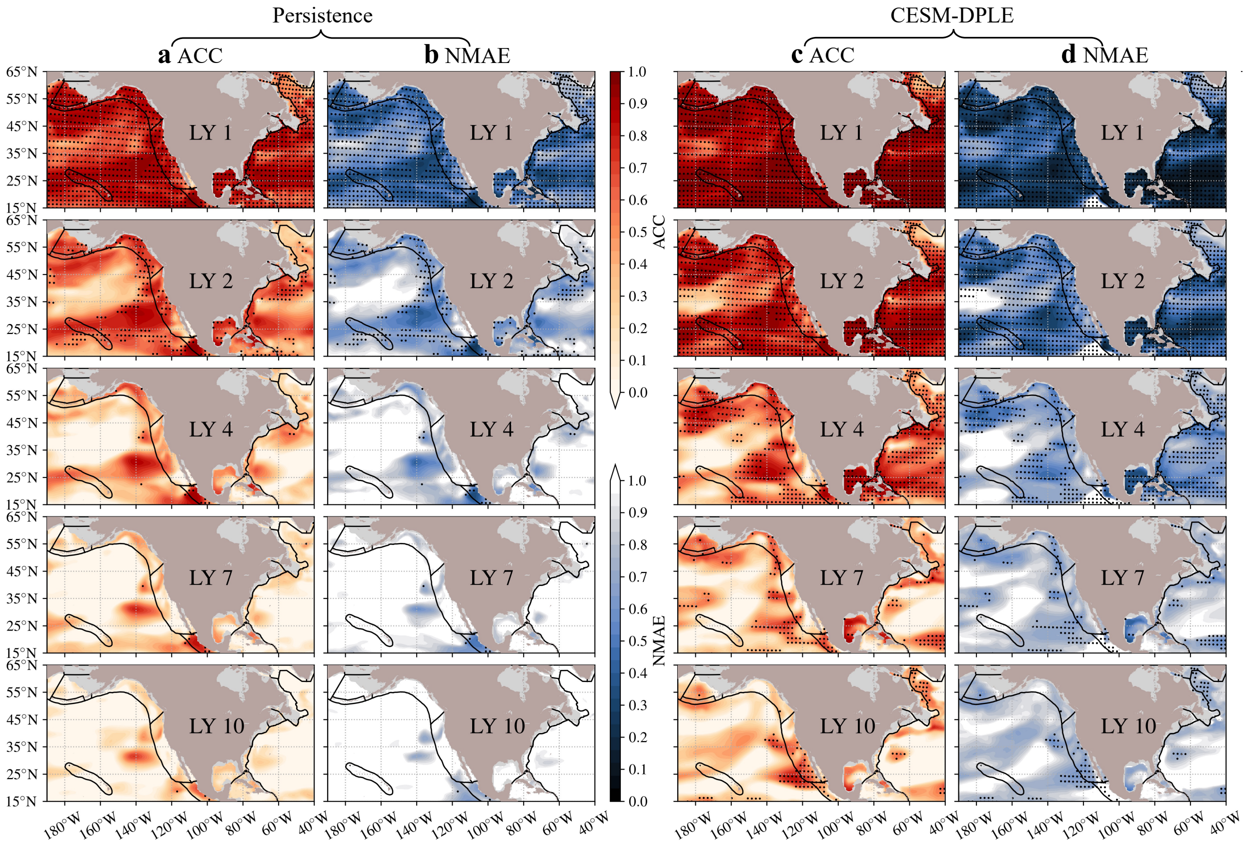

Supplementary Figure 3. Same as Figure 3, but for the deeper 200-600 m layer. Source data are provided as a Source Data file.

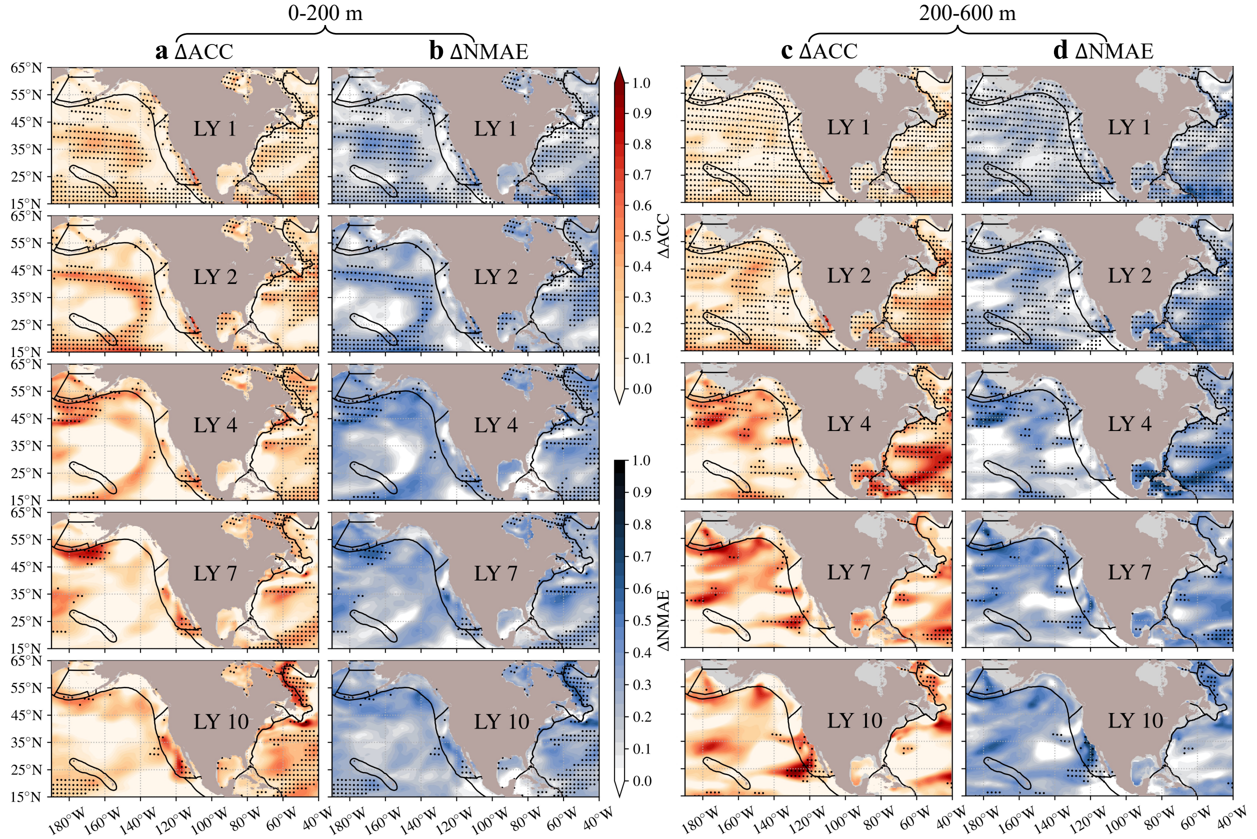

Supplementary Figure 4. Same as Figure 3 and Supplementary Figure 3, but for the spatial skill improvement in Anomaly Correlation Coefficient (ACC) and Normalized Mean Absolute Error (NMAE) of the Decadal Prediction Large Ensemble (DPLE) forecast against the simple persistence forecast at lead year (LY) 1, 2, 4, 7, and 10. The ACC difference (ΔACC) is calculated as the DPLE ACC minus persistence ACC. The NMAE difference (ΔNMAE) is calculated in the opposite manner (persistence minus DPLE). The black dots in each panel indicate the DPLE ACC significantly increased (ΔACC>0) from that of persistence at the 95% confidence level. Source data are provided as a Source Data file.

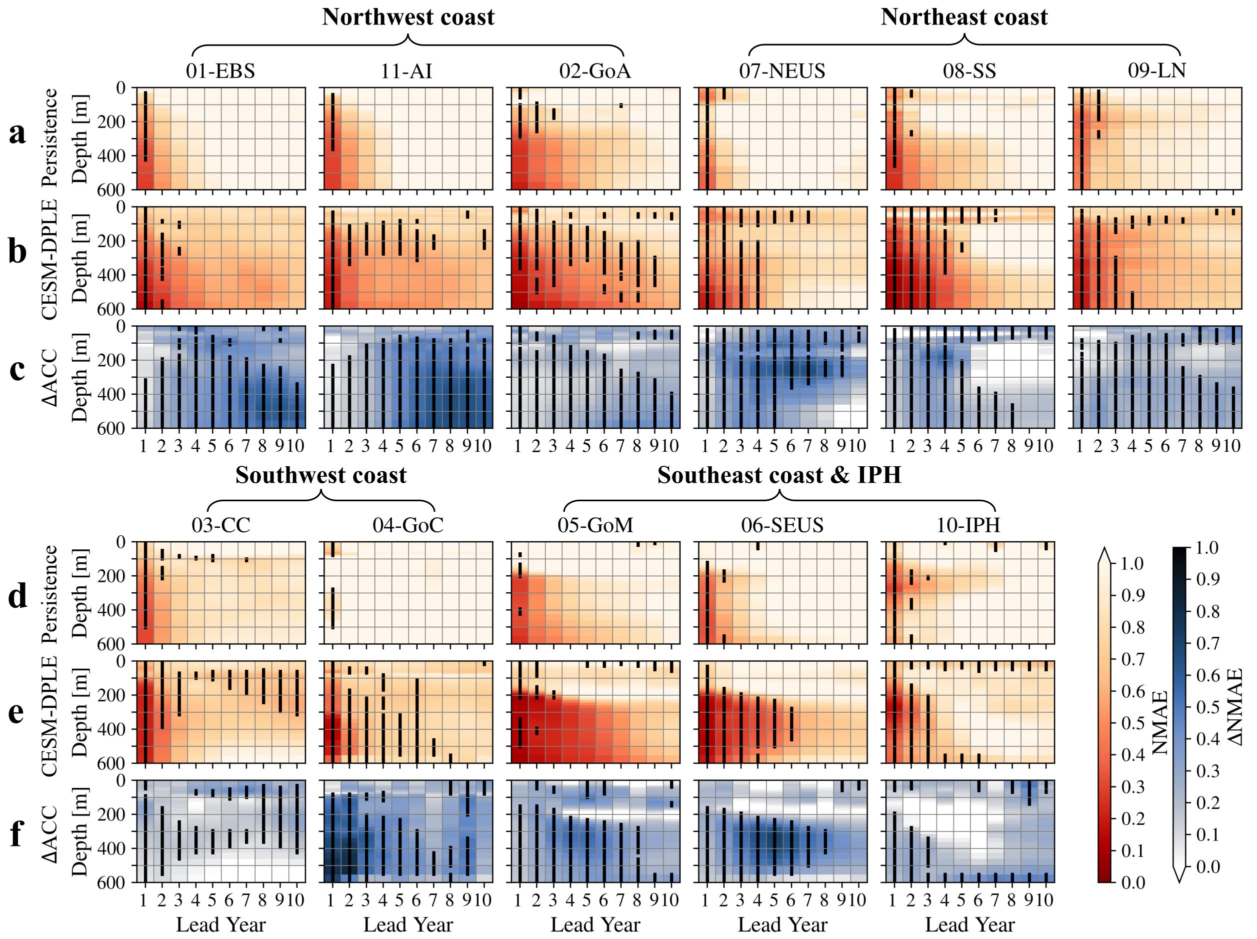

Supplementary Figure 5. Same as Figure 4, except the color shading in each panel represents the corresponding prediction skills of the Normalized Mean Absolute Error (NMAE; **a-b** and **d-e**) and the improved skills from the Decadal Prediction Large Ensemble (DPLE) forecast relative to persistence (ΔNMAE; persistence minus DPLE; **c** and **f**). Source data are provided as a Source Data file.

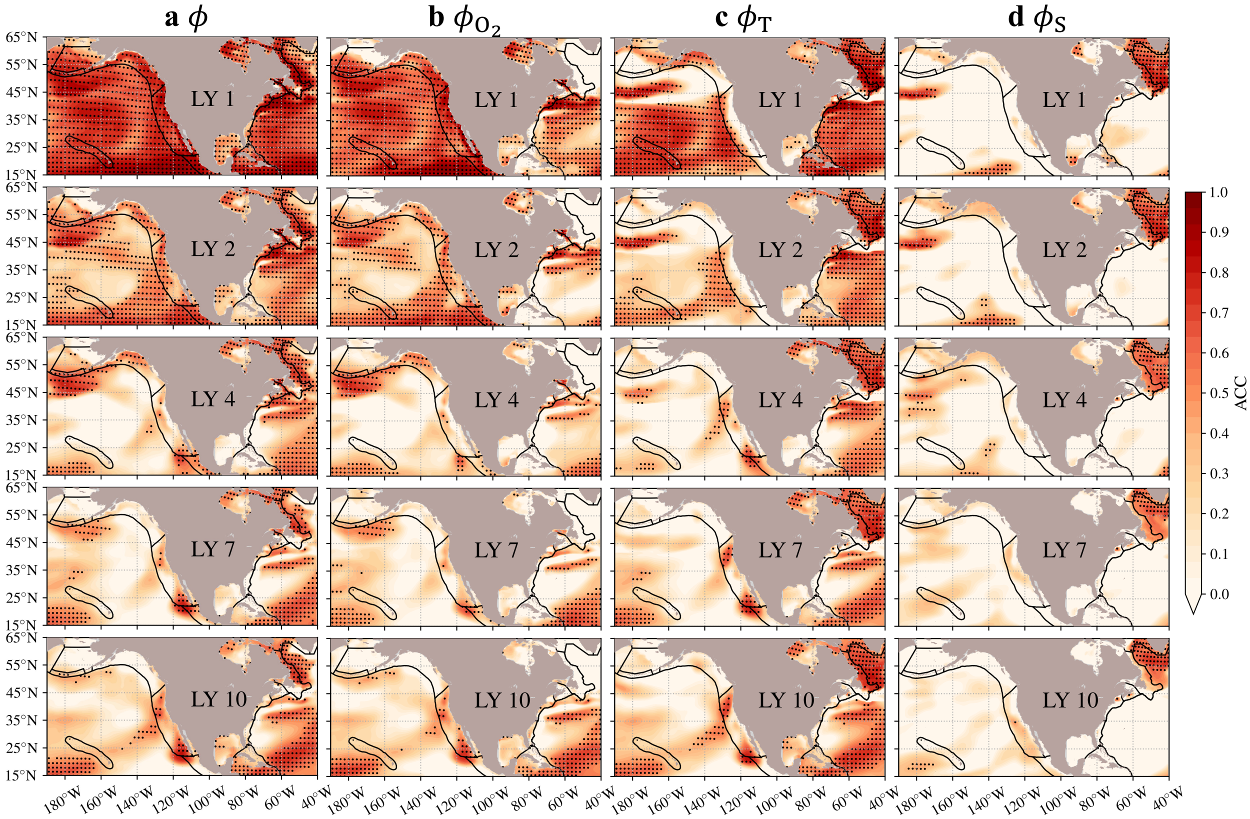

Supplementary Figure 6. Spatial comparison of the interannual-to-decadal prediction skills between the normalized Metabolic Index (ϕ; **a**) and its oxygen (ϕ_{O_2}; **b**), temperature (ϕ_T; **c**), and salinity (ϕ_S; **d**) components, at lead years of 1, 2, 4, 7 and 10. The prediction skills are assessed as the Anomaly Correlation Coefficient (ACC) using the decadal prediction system at the upper 200 m layer. Source data are provided as a Source Data file.

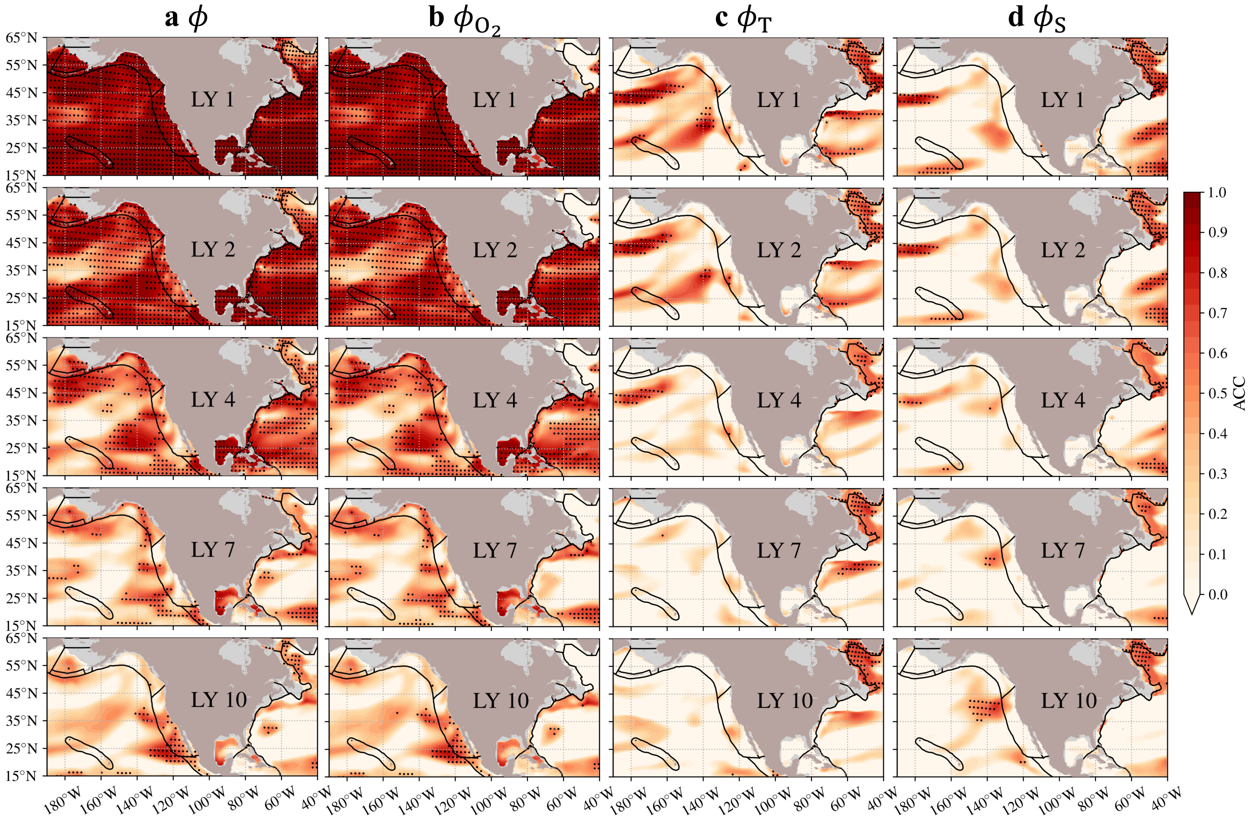

Supplementary Figure 7. Same as Supplementary Figure 6, but for the deeper 200-600 m layer. Source data are provided as a Source Data file.

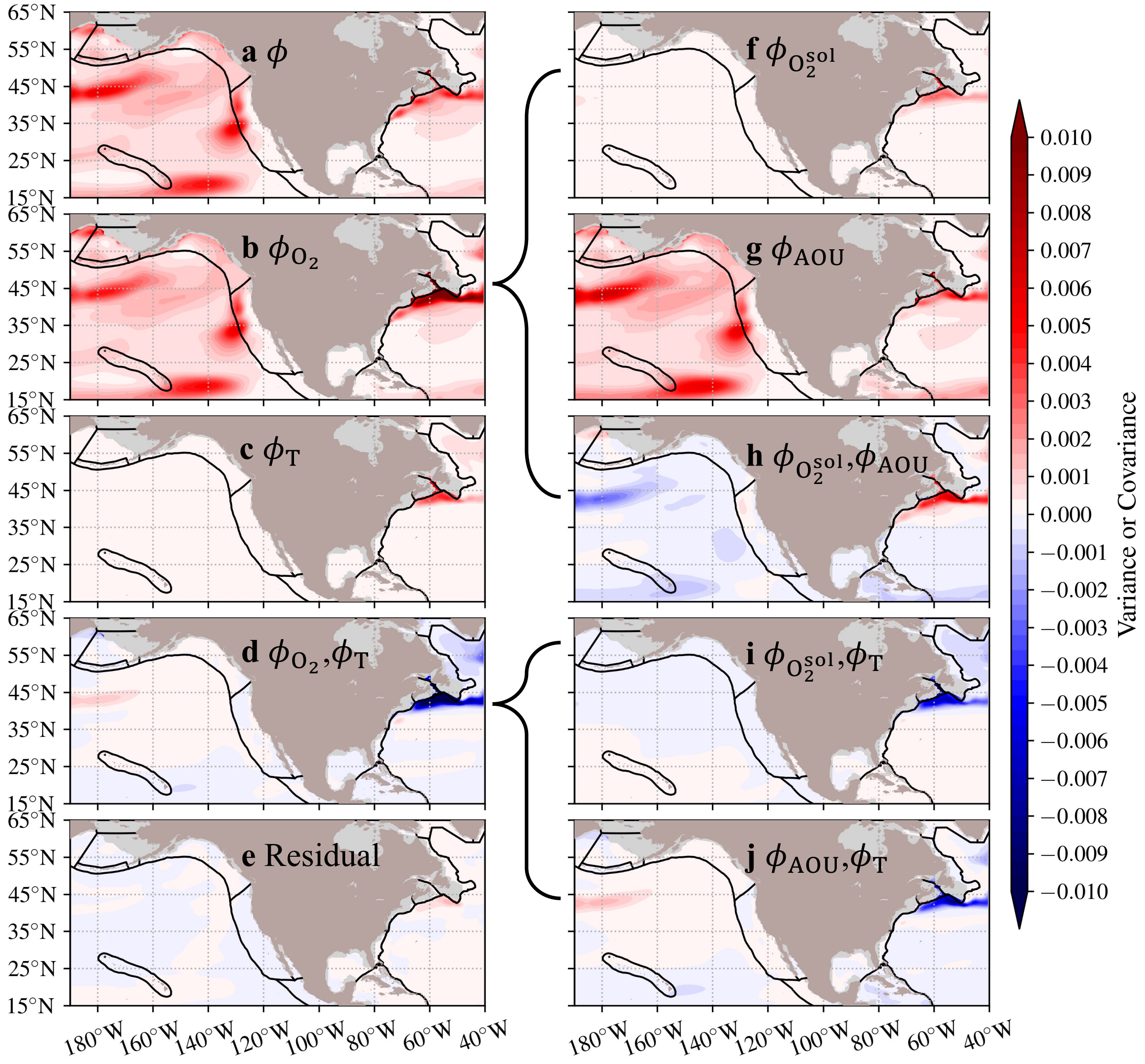

Supplementary Figure 8. Same as Figure 6, but for the deeper 200-600 m layer. Source data are provided as a Source Data file.

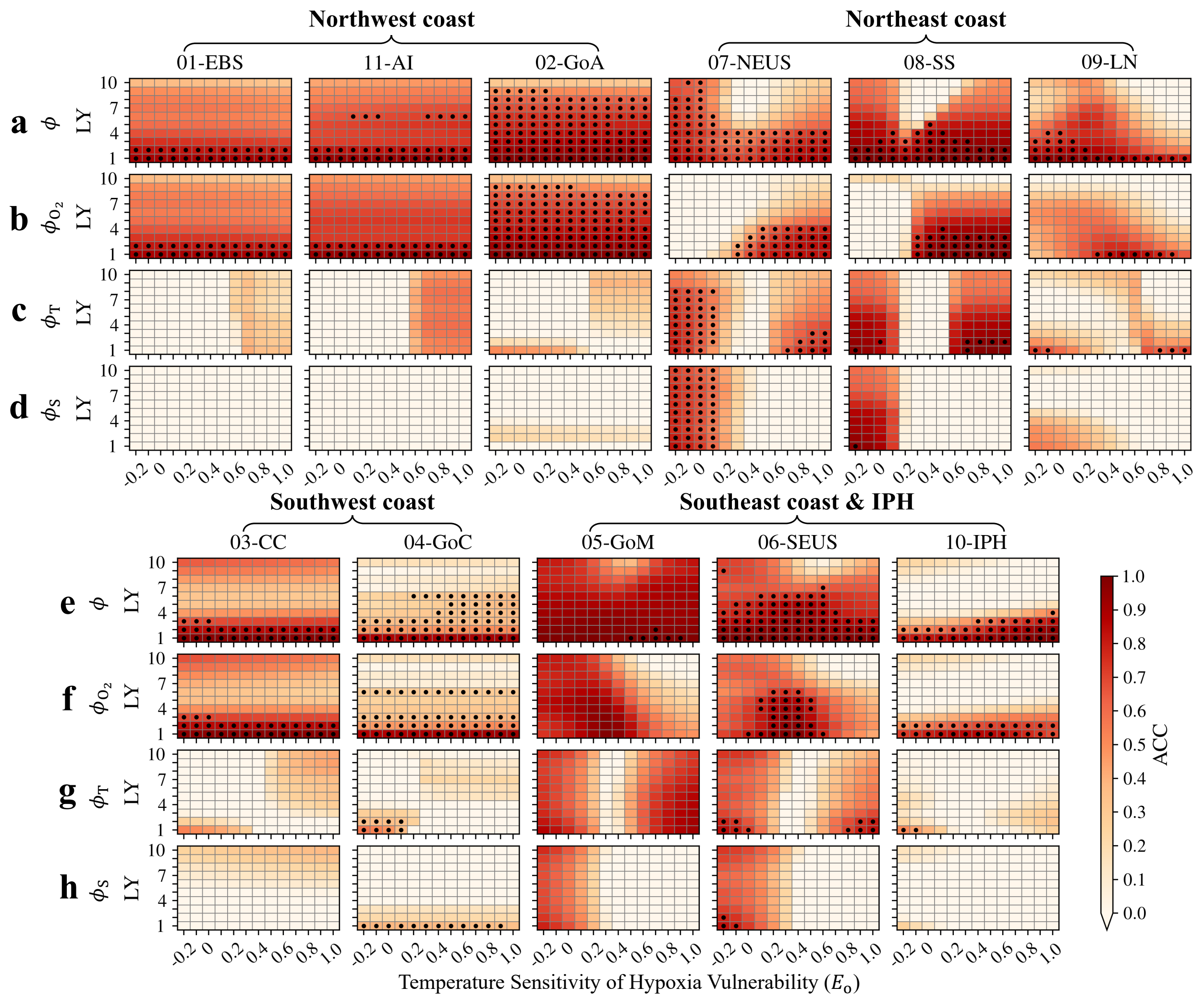

Supplementary Figure 9. Same as Figure 7, but for the deeper 200-600 m layer. Source data are provided as a Source Data file.

Supplementary References

1. Deutsch, C., Penn, J.L. & Seibel, B. Metabolic trait diversity shapes marine biogeography. *Nature* **585**, 557-562 (2020).
2. Sharp, J. D., Fassbender, A. J., Carter, B. R., Johnson, G. C., Schultz, C., & Dunne, J. P. GOBAI-O2: A Global Gridded Monthly Dataset of Ocean Interior Dissolved Oxygen Concentrations Based on Shipboard and Autonomous Observations (NCEI Accession 0259304). NOAA National Centers for Environmental Information. https://doi.org/10.25921/z72m-yz67 (2022).
3. Good, S. A., Martin, M. J. & Rayner, N. A. EN4: Quality controlled ocean temperature and salinity profiles and monthly objective analyses with uncertainty estimates. *Journal of Geophysical Research: Oceans* **118**, 6704-6716 (2013).
